# Long non-coding RNA CRNDE promotes gallbladder carcinoma carcinogenesis and as a scaffold of DMBT1 and C-IAP1 complexes to activating PI3K-AKT pathway

**DOI:** 10.18632/oncotarget.12023

**Published:** 2016-09-14

**Authors:** Sheng Shen, Han Liu, Yueqi Wang, Jiwen Wang, Xiaolin Ni, Zhilong Ai, Hongtao Pan, Houbao Liu, Yebo Shao

**Affiliations:** ^1^ Department of General Surgery, Zhongshan Hospital, Fudan University, Shanghai 200032, China

**Keywords:** Deleted in malignant brain tumors 1 (DMBT1), CRNDE, c-IAP1, migration and invasion, GBC carcinogenesis

## Abstract

Deleted in malignant brain tumors 1 (DMBT1) is deleted during cancer progression and as a potential tumor-suppressor gene in various types of cancer. However, its role in Gallbladder cancer remains poorly understood. DMBT1 has low-expression and deletion of copy number were detected in normal tissues and GBC cancer tissues by qRT-PCR. Knockdown of DMBT1 increased migration and invasion and overexpressed DMBT1 impaired migration and invasion in GBC cells. We also evaluated the molecular mechanism of DMBT1 by RNA sequencing and GSEA analysis. RNA-Pulldown and RIP assay authenticated CRNDE can specified binding with DMBT1 and c-IAP1. Downregulation of DMBT1 resulted in significant change of gene expression (at least 2-fold) in PI3K-AKT pathway, increased expression of MMP-9, JUK-1, ERK and AKT, activating PI3K-AKT pathway lead to GBC carcinogenesis.

We for the first time reported, DMBT1 as a prognosis biomarker, is low-expressed in GBC tumors, and CRNDE act as a scaffold to recruit the DMBT1 and c-IAP1, promotes the PI3K-AKT pathway. Our study reveals DMBT1 may be an important contributor to GBC cancer development.

## INTRODUCTION

Gallbladder cancer (GBC), first described in 1777, is the most malignancy of the biliary tract and the fifth death-leading in gastrointestinal cancer worldwide [1–3]. The prognosis of GBC remains extremely poor despite recent advances in GBC treatment, with a median survival time of 10 months for suspected carcinomas and two years for incidental GBC [4, 5]. Thus, it is vital to reveal its pathogenesis and understanding molecular mechanisms of gallbladder carcinogenesis to facilitate development of novel cancer biomarkers and more effective therapeutic strategies.

Deleted in Malignant Brain Tumors 1 (DMBT1) was identified the extracellular secreted scavenger receptor cysteine-rich protein, located in chromosome 10q25.3-q26.1, with predominant expression in the intestine [6]. Several studies showed DMBT1 have a function of tumor-suppressor based on homologous deletions and low expression in various kinds of cancers with function in immunity, inflammation and epithelial cell differentiation [7–9]. Studies showed DMBT1 also a target of TLR4 through NOD2 which promote IBD susceptibility [10, 11]. In spite of its function in proliferation, migration and adhesion of endothelial cells in previous studies [12, 13]. Yet, there have been no systematic profiling studies of DMBT1 in gallbladder cancer up until now.

Increasing studies have focused on long non-coding RNAs (lncRNAs), which is a length of more than 200 nucleotides and without protein-coding potential are located both in nuclear or cytoplasmic. Mounting evidence has shown that LncRNAs frequently transcribed by RNA polymerase II without open reading frame, participate in many biological processes include cell proliferation, apoptosis, invasion [14–16] chromosome imprinting and histone modification [17–19].

Colorectal Neoplasia Differentially Expressed (CRNDE)[20], which is located on chr16:54,952,777–54,963,101, is the most up-regulated lncRNA in colorectal cancer [21]. CRNDE, shares bi-directional promoter with iroquois homeobox 5 (IRX5), which is adjacent at the opposite strand, have been demonstrated over-expressed in many specific regions of human brain, and also upregulated in gliomas [22]. However, the function and molecular mechanism of CRNDE in GBC remains unclear.

In the present study, we found DMBT1 has lower expression in 96 paired cDNA of GBC and normal gall bladder tissues, with deleted copy numbers in 42 paired DNA of GBC and normal gall bladder tissues. *In vitro* and *in vivo* assays, we demonstrated the function of DMBT1 on invasion and migration. By RNA-seq and GSEA analysis, we found DMBT1 can directly binds with CRNDE/ c-IAP1 to promote PI3K-AKT pathway.

Our study might offer a novel prognostic indicator in GBC and explore the feasibility of dysregulated lncRNA-protein binding oriented diagnosis and gene therapy for this deadly disease.

## RESULTS

### Low DMBT1 expression is associated with the prognosis of GBC

**Figure 1:**
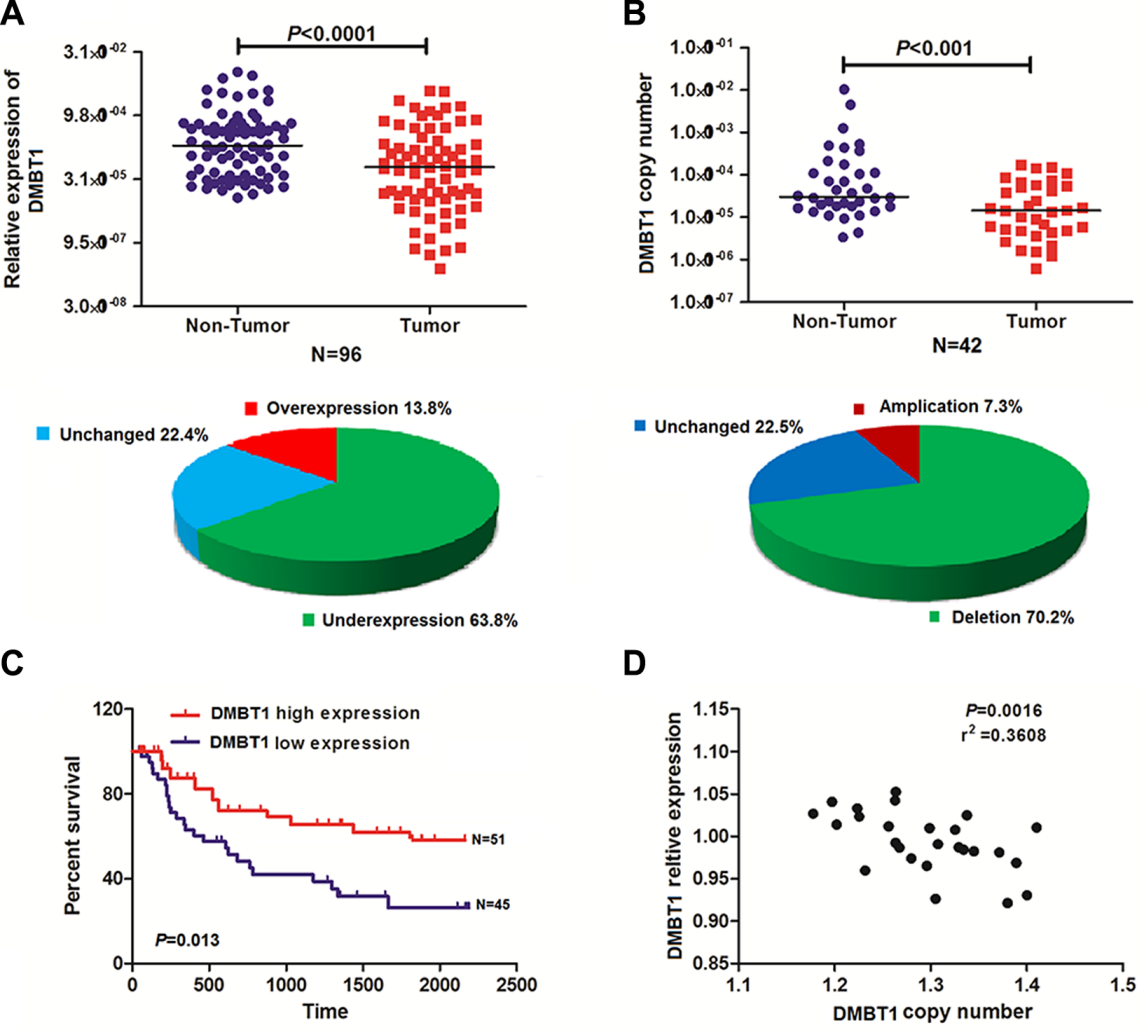
DMBT1 expression was frequently reduced in GBC as a result of DNA copy number variation (**A**) Comparison of DMBT1 expression levels in 96 matched pairs of GBC tissues and non-tumor tissues (NTs). The qRT-PCR results showed that DMBT1 expression was significantly downregulated in tumor tissues. DMBT1 expression was upregulated in 13.8%, downregulated in 63.8% and unchanged in 22.4% of the GBC samples. (**B**) DMBT1 copy numbers were determined for 42 matched GBC tissues and non-tumor tissues (NTs) using qPCR. The copy number of DMBT1 was decreased in the tumor tissues compared with the NCTs. The copy numbers of DMBT1 DNA were increased in 7.3%, decreased in 70.2% and unchanged in 22.5% of the GBC samples compared with NTs. (**C**) Difference in survival between high level group and low level group was analyzed by the log-rank test. Patients in high- DMBT1 group showed decreased overall survival (OS, A), as compared with low- DMBT1 group, *P* = 0.013. (**D**) DMBT1 expression was positively correlated with the DMBT1 DNA copy number in both the GBC samples and the NT samples.

DMBT1 mRNA expression in control gall bladder and GBC cancer tissues was evaluated by qRT-PCR and normalized to an internal control (β-actin). Expression of DMBT1 in GBC tissues is significantly lower when compared to the corresponding Non-Tumors (63.8%) (*P* < 0.001, Figure 1A). To confirm the hypothesis that downregulation of DMBT1 in GBC tissues was partly caused by copy number variation of its coding sequence, we determined the DMBT1 copy number by qRT-PCR in 42 pairs of the GBC tissues and Non-Tumors. Interestingly, we found that the copy number of DMBT1 was lower in 70.2% and higher in 7.3% of the DMBT1 tissues compared with their Non-Tumors counterparts (*P* < 0.001, Figure 1B). Subsequent analysis showed that the DMBT1 copy numbers have positive related to the expression levels in both the GBC and Non-Tumors samples (*P* < 0.05, Figure 1D).

To explore whether DMBT1 expression was associated with the poor prognosis of GBC cancer, analysis was used to assess the expression level of DMBT1 in 96 pairs of GBC tissues and matched normal tissues. The clinical pathological characteristics of the 96 patients with GBC are summarized in Supplementary Table S1. The correlation analysis of DMBT1 expression revealed a significant association between DMBT1 expression and GBC tumor size (*P* < 0.01), tumor stage (T stage) (*P* < 0.01), node stage (N stage) (*P* < 0.01), and increased death (*P* < 0.01) (Supplementary Table S1). However, the association between DMBT1 expression and patient age, gender, and grade was not found. Kaplan–Meier survival analysis and log-rank tests revealed that patients with high DMBT1 expression had a significantly longer overall survival (OS) than those with low DMBT1 expression (Figure 1C, *P* < 0.01). Univariate and multivariable Cox regression analyses revealed that grade and high DMBT1 expression were risk factors for the OS of patients with GBC (Supplementary Table S2–S3). These results indicated that DMBT1 expression could be an independent factor for increasing the OS.

These data suggested that lower level of DMBT1 expression was associated with GBC cancer progression. Thus, consistent with tumor suppressor function, decreased expression of DMBT1 mRNA occurs frequently in GBC.

### DMBT1 suppresses GBC cell migration and invasion both *in vivo* and *in vitro*

**Figure 2:**
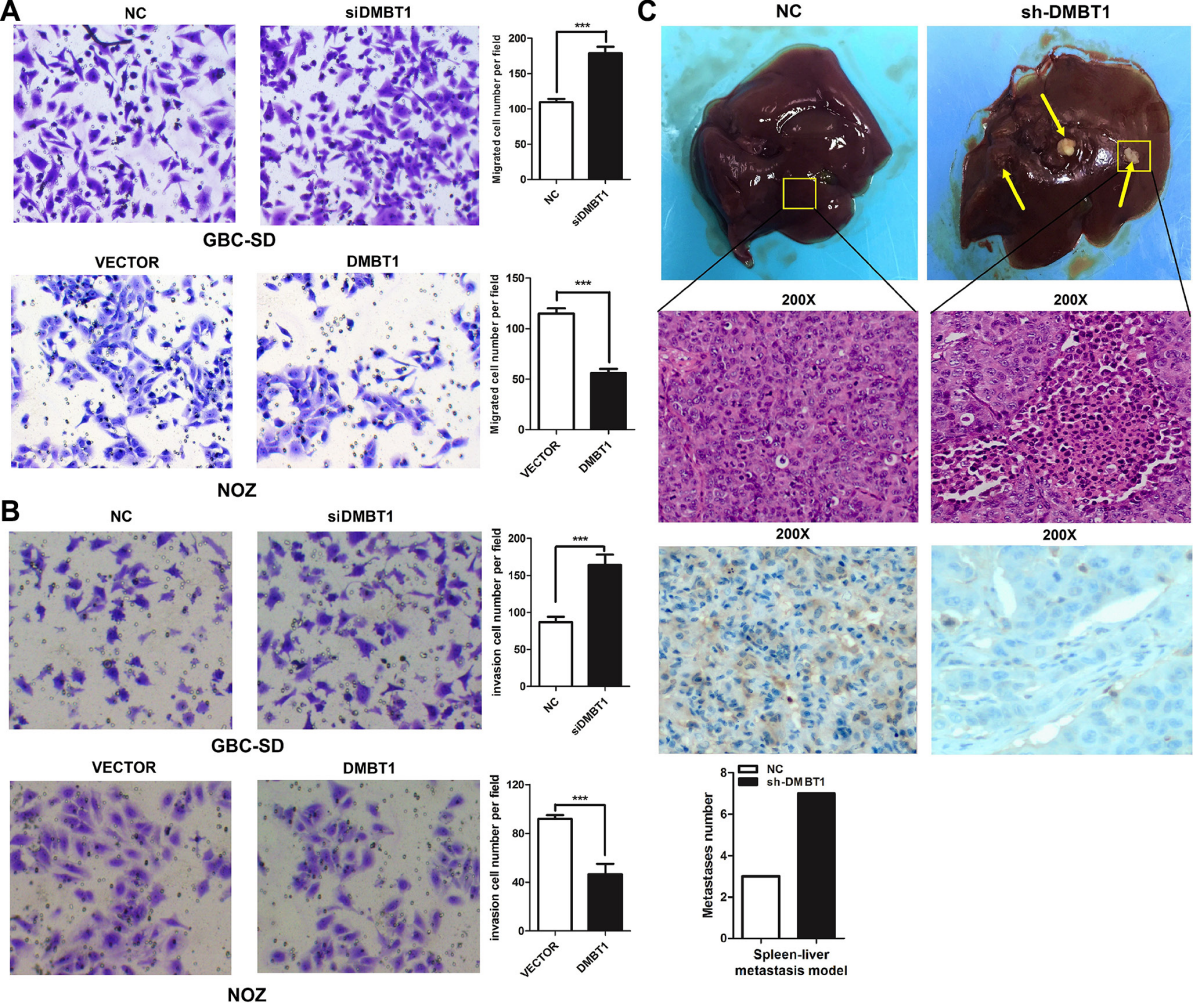
DMBT1 represses GBC cell migration and invasion *in vitro* and *in vivo* (**A**–**B**) Knockdown of DMBT1 enhanced the GBC-SD and NOZ cell migration and invasion. DMBT1 overexpression represses the migration and invasion of GBC-SD and NOZ cell. The migration and invasion number of cells was evaluated by crystal violet staining and counted (***P < 0.001). (**C**) The effect of DMBT1 on tumor invasion in a nude mouse xenograft model. Lentiviral vector- and DMBT1-infected GBC-SD cells (1 × 10^7^) were injected into the nude mouse. The tumor invasion node of the si-DMBT1 group was significantly decreased compared with the control group (***P < 0.001). Representative Photomicrograph of HE-stained and ISH characteristics of DMBT1 expression are shown (magnification, left panel, ×100; right panel, ×200).

To investigate the functional effects of DMBT1 in GBC cells, we constructed two short interfering RNAs (siRNAs) targeting DMBT1 and transfected into GBC-SD and NOZ cells. Therefore, we used this siRNA in all the following experiments. To further assess the potential effects of RNAi-mediated DMBT1 silencing on cell migration, transwell assay was performed after si-DMBT1 transfection into GBC cells. Compared with si-NC transfected cells, a significant increase of cell number was found in both GBC-SD and NOZ cells after treatment with si-DMBT1 (Figure 2A–2B). DMBT1 was highly expressed by transfected by PWPXL-DMBT1 and upregulation of DMBT1 decreased the migration and invasion of GBC-SD and NOZ GBC cell lines. The results of transwell assay showed that the silencing of DMBT1 could significantly improve GBC cell invasion ability compared with control cells and that the PWPXL-DMBT1 could impaired the ability.

Next, the effects of DMBT1 on invasion *in vivo* were investigated. A tumor invasion assay in a nude mouse model was performed using GBC-SD cells stably sh-DMBT1 after lentiviral infection. Compared with the empty vector control, knockdown of DMBT1 remarkably promoted tumorigenesis in nude mice (*P* < 0.01, Figure 2C). These results showed DMBT1 as a cancer suppressor gene in GBC.

### DMBT1 directly binds with CRNDE/ c-IAP1 in GBC cancer cells

**Figure 3:**
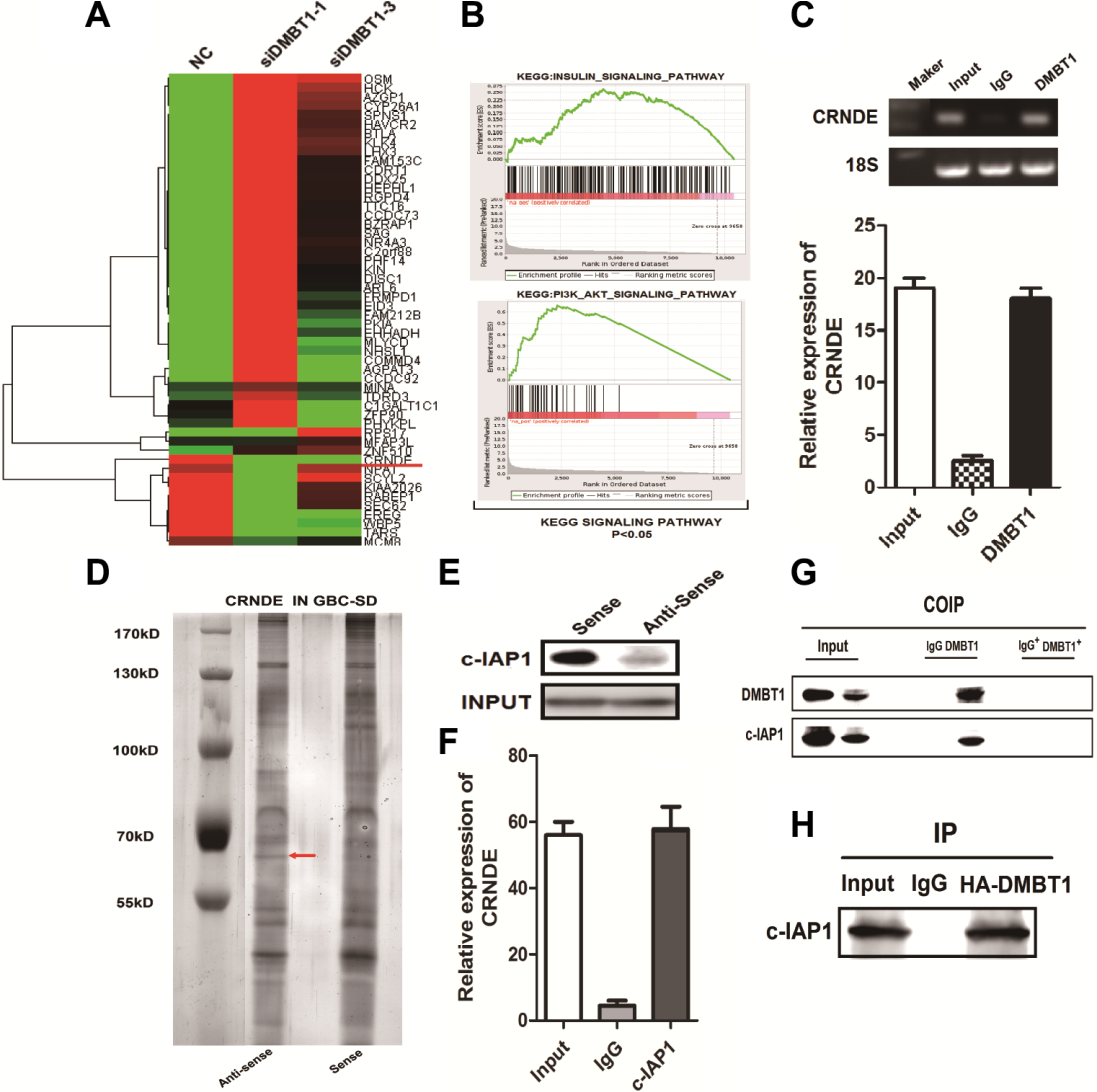
DMBT1 bound to CRNDE and C-IPA1 (**A**–**B**) Heat map for the Relative expression of si-DMBT1 and si-NC in GBC-SD cells analyzed by using GSEA datasets and bioinformatics predictions. (**C**) RIP analyses were performed using antibodies against DMBT1, with IgG as a negative control in GBC-SD cells. The enrichment of the CRNDE was detected using RT-PCR and normalized to the input. (**D**) RNA pull-down was performed using a CRNDE template and RNA-binding protein separated by SDS-PAGE in GBC-SD cells. The protein bands were excised and detected by mass spectrometry analysis. (**E**–**F**) C-IAP1 was detected by the Western blotting assay in the samples pulled down by CRNDE. RIP analyses were performed using antibodies against c-IAP1, with IgG as a negative control in GBC-SD cells. The enrichment of the CRNDE was detected using RT-PCR and normalized to the input. (**G**–**H**) Co-IP assay detected by western blot. IgG as a negative control in GBC-SD cells.

To elucidate whether DMBT1 plays a role in GBC tumorigenesis, a RNA-seq analysis was performed to compare the gene expression profiles of DMBT1 siRNA and control si-NC transfectants (Figure 3A). Gene set enrichment analysis (GSEA) revealed that the gene sets related to INSULIN_SIGNALING_PATHWAY and PI3K_AKT_SIGNALING_PATHWAY (Figure 3B). Several recent studies have shown that many lncRNAs could participate in molecular regulation pathways via their binding with proteins. And we found a known lncRNA-CRNDE was significantly high expression when knockdown of DMBT1. To substantiate the observation, anti-DMBT1 antibody was used to immunoprecipitate edogenous DMBT1 in GBC-SD cells. And RNAs bound to DMBT1 were extracted and analyzed. PCR data revealed that CRNDE directly bound with DMBT1 in GBC cancer cells (Figure 3C). LncRNAs may function by physically interacting with transcriptional factors, histone regulators and other cellular protein factors. To investigate whether CRNDE functioned in a similar manner, RNA pull-down assays were performed to identify proteins associated with CRNDE in GBC-SD cancer cells (Figure 3D). Baculoviral IAP repeat containing 2 (c-IAP1) was the main proteins identified by mass spectrometry (Supplementary Excel S1), and both of them were detected by Western blotting in three independent RNA pull-down assays (Figure 3E). RIP was also performed using anti-c-IAP1 (IgG for control) in cell extracts from GBC-SD cells. CRNDE enrichment with c-IAP1 antibodies was observed, but not GAPDH mRNA enrichment (Figure 3F). These results suggested that CRNDE probably physically bound with both c-IAP1 and DMBT1.

To explore the relationship between DMBT1 and c-IAP1, we performed IP and Co-IP assay between them. Results showed in Figure 3G–3H illustrated DMBT1 and c-IAP1 interacted with each other in GBC-SD cells.

These results suggested that c-IAP1 and DMBT1 were involved in the effects of CRNDE, and partly via INSULIN_SIGNALING_PATHWAY and PI3K_AKT_SIGNALING_PATHWAY.

These findings suggested that CRNDE may act as a scaffold for this complex, thereby controlling its ability to regulate the biological activity.

### CRNDE, stabilized by DMBT1, which can stabilizing the protein of c-IAP1

**Figure 4:**
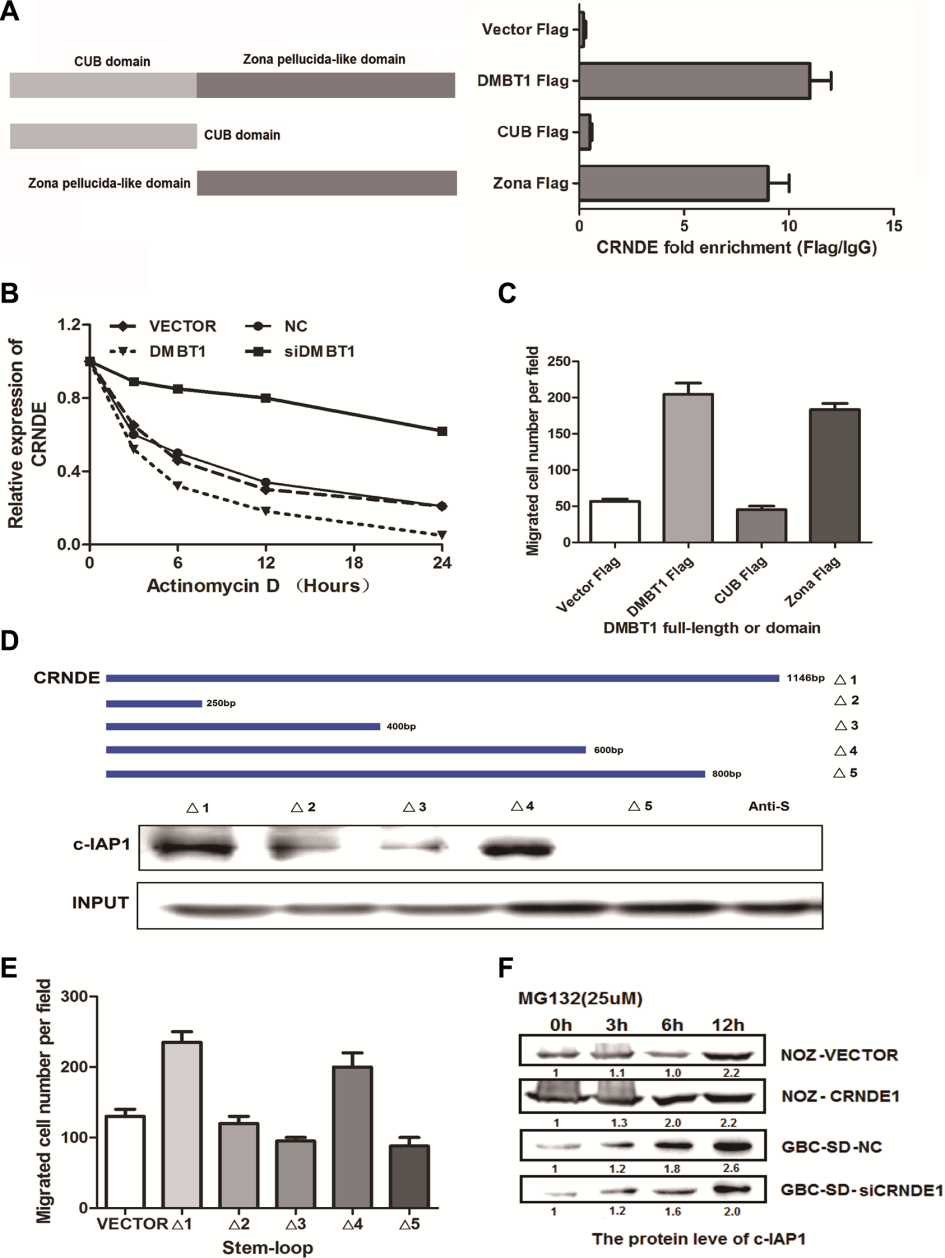
The binding form in DMBT1-CRNDE-c-IAP1 complex (**A**) Deletion mapping of the DMBT1 domain in binding with CRNDE. RIP assays show association of deletion-DMBT1 domain with CRNDE in GBC-SD cells. Relative enrichment (means ± s.e.m.) represents RNA levels associated with domains relative to an input control from three independent experiments. Antibodies against FLAG and control IgG served as controls. (**B**) GBS-SD cells with stable overexpression of DMBT1 or knockdown of DMBT1 were treated with Actinomycin D (2.5 μM) for 24 h. Detect the mRNA level by RT-PCR. (**C**) Migration numbers of transwell assay of different deletion mapping of the DMBT1 domains. (**D**) Deletion mapping of the C-IAP1-binding domain in CRNDE. Top, diagrams of full-length CRNDE and the deletion fragments. Middle, the *in vitro*–transcribed full-length CRNDE and deletion fragments show the correct sizes. Bottom, immunoblot analysis for C-IAP1 in protein samples pulled down by the different CRNDE constructs. (**E**) Migration numbers of transwell assay of different deletion mapping of the CRNDE fragments. (**F**) GBS-SD cells with stable overexpression of CRNDE or knockdown of CRNDE were treated with MG132 (5 μM) for 24 h. Cell lysates were immunoprecipitated with antibody against C-IAP1. The precipitates and input were analyzed by immunoblotting.

To explore the mechanism of DMBT1-CRNDE- c-IAP1 in GBC cells, we performed the mapping assay of them. In Figure 4A, we constructed two main domain of DMBT1 in FLAG-labeled mutant proteins which named FLAG-CUB and FLAG-Zona. RIP assay showed CRNDE specified binding with DMBT1 in FLAG-Zona area. And FLAG-Zona domain play an important function in migration (Figure 4C). To explore the mechanism between DMBT1 and CRNDE, An actinomycin D treated assay showed DMBT1 can stabilized the CRNDE in mRNA level (Figure 4B). To identify the c-IAP1-interacting region of CRNDE, we constructed and biotinylated five fragments of CRNDE (Δ1:1-1146 bp; Δ2: 1-250 bp, Δ3 : 1-400 bp, Δ4 : 1-600 bp, Δ : 1-800 bp), and used them in the pull-down assay with GBC-SD cell lysates. We found that the 5′ fragment of CRNDE (Δ4 : 1-600bp) mediated the interaction with c-IAP1(Figure 4D). And Δ4 fragment play an important function in migration of GBC cells (Figure 4E). And, MG132 treated assay showed CRNDE can stabilized the protein level of c-IAP1. Taken together, these results illustrated the interactions among DMBT1, CRNDE and c-IAP1.

### C-IAP1 and CRNDE were regulated by DMBT1 in GBC cancer

**Figure 5:**
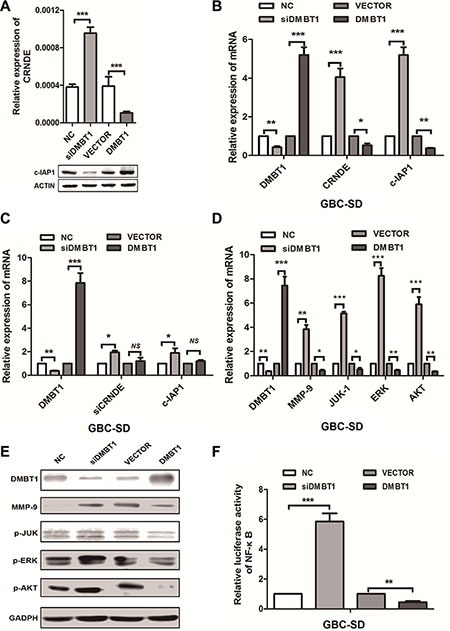
DMBT1 regulate CRNDE and c-IAP1 through PI3K-AKT pathway (**A**) RT-qPCR and western blot were used to detect the mRNA levels of CRNDE and protein level of c-IAP1 in knockdown and overexpression DMBT1 cells. (**B**) RT-qPCR was used to detect the mRNA levels of CRNDE and c-IAP1 in knockdown and overexpression DMBT1 cells. (**C**) RT-qPCR was used to detect the mRNA levels of CRNDE and c-IAP1 in knockdown and overexpression DMBT1 cells when knockdown of CRNDE. (**D**) Validation of the RNA-seq results in GBC-SD cells using qRT-PCR. A panel of 4 genes were indeed up regulated by knockdown of DMBT1. (**E**) Four candidate genes (MMP-9, P-JUK, P-ERK, and P-AKT) were selected for western blot analysis. (**F**) luciferase activity of NF-κB pathway in DMBT1-transfected cells was increased, and decreased when overexpression of DMBT1.

We next explored the mechanism by which DMBT1 affect the expression of CRNDE and c-IAP1. Realtime-PCR and western blot data showed that knockdown of DMBT1 can increase the mRNA of CRNDE and decrease the protein level of c-IAP1. And overexpression of DMBT1 can decrease the expression of CRNDE and increase the protein level of c-IAP1 (Figure 5A). Also, we detected the mRNA level of CRNDE and c-IAP1 when knockdown and over-expression DMBT1, result in Figure 5B showed, when we knockdown of DMBT1, the mRNA level of CRNDE and c-IAP1 have significantly increased, and when overexpression of DMBT1 mRNA level of CRNDE and c-IAP1 were largely decreased. On the contrary, when we knockdown of CRNDE in knockdown DMBT1 cells, the mRNA level of c-IAP1of GBC largely decreased. In overexpression DMBT1, show the non-sense changes (Figure 5C). These results declare the DMBT1 affect the expression level of c-IAP1 and CRNDE in the different level.

### DMBT1 suppress the migration and invasion by regulates the PI3K-AKT pathway in GBC

As for the RNA-seq results, we select top-scoring genes recurring in PI3K-AKT pathway include MMP-9, JUK-1, ERK and AKT. We found when knockdown of DMBT1, the mRNA and protein level of MMP-9, JUK-1, ERK and AKT have significantly increased. And in overexpression DMBT1 group, the mRNA and protein level of these genes were decreased (Figure 5D–5E). Besides, we performed the luciferase assay (Figure 5F), when knockdown of DMBT1, the Luc-activity of NF-κB pathway was increase, on the contrary, the Luc-activity of NF-κB pathway in overexpression of DMBT1 was decrease. These results showed DMBT1 can regulates the PI3K-AKT pathway by influence the MMP-9, JUK-1, ERK and AKT, to suppress the migration and invasion of GBC cancer cells.

### CRNDE had a positive correlation with c-IAP1 in GBC

**Figure 6:**
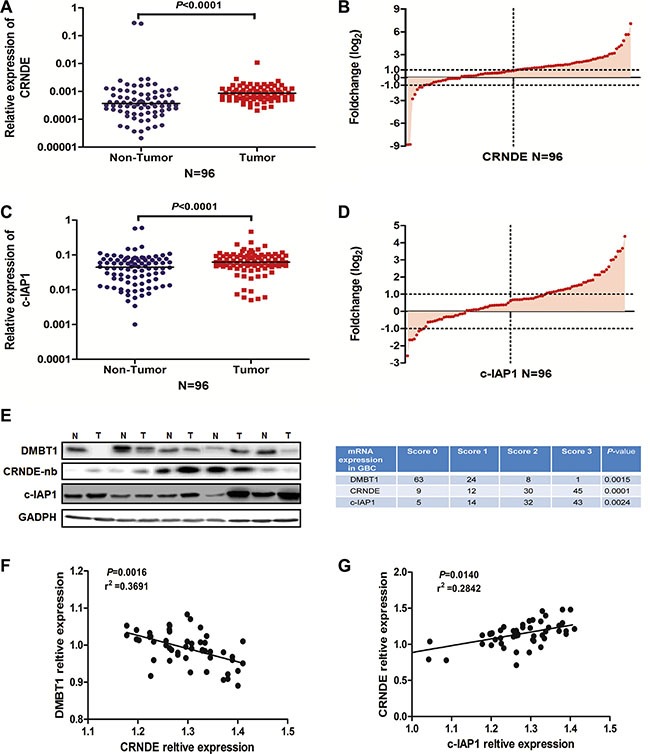
The positive correlation between CRNDE and c-IAP1 (**A**–**B**) RT-qPCR was used to detect the mRNA levels of CRNDE in 96 paired GBC tissues. CRNDE significantly upregulated in tumor tissues (β-actin used as an internal control). (**C**–**D**) RT-qPCR was used to detect the mRNA levels of c-IAP1 in 96 paired GBC tissues. c-IAP1 significantly upregulated in tumor tissues (β-actin used as an internal control). (**E**) Western blot and northern blot assay to detected the DMBT1, C-IAP1 and CRNDE expression level in five paired GBC tissues and NT tissues. (**F**) Correlation between DMBT1 and CRNDE in 42 paired GBC tissues. (**G**) Correlation between CRNDE and c-IAP1 in 42 paired GBC tissues.

In previous studies, CRNDE expression is significantly up-regulated in a number of neoplastic diseases, including colorectal cancer [21]. More strikingly, the increased CRNDE expression observed in cancers always accompanying abnormal expression of related-genes in cell types or tissues, suggesting that CRNDE may be a key modulator in tumorigenesis. So we detected the expression level of CRNDE and c-IAP1 in 96 paired of GBC tissue, results in Figure 6A–6D showed they have a high expression in GBC tissue. Also we detected the protein level of DMBT1 and c-IAP1 in GBC tissues by western blot, detected the CRNDE mRNA level in GBC tissues by northern blot. Results (Figure 6E) showed c-IAP1 (*P* = 0.0024) and CRNDE (*P* = 0.0001) had a higher expression level in GBC tissue; and DMBT1 (*P* = 0.0015) had a lower expression in GBC tissue. At last, we detected the correlation between DMBT1 and CRNDE, CRNDE and c-IAP1, results showed DMBT1 and CRNDE had a negatively correlation and CRNDE and c-IAP1 had a positive correlation in GBC tissues. Which confirmed the results we done above. These results illustrated CRNDE may act as scaffold of DMBT1 and c-IAP1 to influence the migration and invasion of GBC cancer cells.

## DISCUSSION

Several studies showed Deleted in malignant brain tumors 1 (DMBT1) have functions in many kinds of cancer [23]. Also, DMBT1 is demonstrated involved in many biological progress include angiogenesis binding with VEGF, cell proliferation of lung cancer [24]. But the study of DMBT1 in GBC cancer remain largely unknown.

An growing interest toward lncRNAs in cancer is sparked, hundreds of lncRNAs have been discovered by RNA-sequencing and emerged as critical regulators of gene expression in both transcriptional and post-transcriptional processes [25]. In addition, many famous lncRNAs have been found to involve in gastric tumorigenesis and progression, such as HOTAIR, H19, MEG3, HOXAAS2 and KRT7-AS, also have functions in another type of tumor [25–27]. Previous studies on CRNDE, focus on its tissue-specific feature with interesting high enriched in testis, breast, and skin, and little expression in adult colorectal mucosa, liver, and white blood cells [28]. Recent study demonstrated that one of the CRNDE transcripts, gVC-In4, contains a highly-conserved sequence within intron 4 and promotes metabolic changes through insulin/IGF signaling in colorectal cancer cells [29]. In this study, we identified, a famous long non-coding RNA, CRNDE, also a key modular in GBC progression.

In our study, we endow the CRNDE a new position, which can specified bind with DMBT1 and C-IAP, leading to promote the GBC progress. Our mass spectrometry and RNA pull-down data have demonstrated that CRNDE directly interacts with the C-IAP1, is a member of a family of proteins that inhibits apoptosis by binding to tumor necrosis factor receptor-associated factors TRAF1 and TRAF2, probably by interfering with activation of ICE-like proteases [30]. C-IAP1 directly bind with CRNDE at the 5′ fragment. And CRNDE can stabilized the protein level of C-IAP1. Knockdown of DMBT1 can increase the mRNA level of CRNDE and c-IAP1, but decease the protein level of c-IAP1. Knockdown of CRNDE disrupts the binding of DMBT1 and C-IAP1 which influence the PI3K-AKT pathway.

Given the clinical, genetic, biochemical, and functional significance of DMBT1 in GBC, we conclude that DMBT1 by binding with CRNDE and c-IAP1 associated with PI3K-AKT pathway is crucial for GBC carcinogenesis, and targeting this pathway may be pivotal in the treatment of GBC.

## MATERIALS AND METHODS

### Clinical specimens and ethics statement

Human tissues samples (96 cases of GBC cancer patients) were from the tissue bank at the Department of Pathology, from Feb 2013 to Nov 2015, Zhongshan Hospital affiliated with the Fudan University (Shanghai, China). In our study, all patients without chemotherapy or radiotherapy before surgery. The Regional Scientific Ethical Committee for Fudan University approved the use of human tissue samples for research purposes and samples were obtained from patients with written informed consent. Normal human gall bladder tissue was obtained from patients undergoing resection for carcinoma of the GBC and samples were taken from regions several centimeters away from the tumor. The procedure was approved by the National Health Service Research Ethics Committee. Human Tissue also used for analysis by SDS-PAGE/Western blotting and immunohistochemistry. All specimens were obtained under sterile conditions during surgery, snap frozen in liquid nitrogen, and stored at −80°C.

### Cell culture

Human GBC cells GBC-SD and NOZ, HEK293T, were obtained from the American Type Culture Collection. Cells were maintained in Dulbecco's Modified Eagles Medium (DMEM) (both from Invitrogen Life Technologies, Carlsbad, CA, USA) supplemented with 10% fetal bovine serum (FBS) (both from Invitrogen Life Technologies, Carlsbad, CA, USA) and 100 units/mL penicillin and 100 μg/mL streptomycin (5% CO2) at 37°C.

### Western blotting and antibodies

Cell protein lysates were extracted from the logarithmically growing cells using radioimmuno- precipitation assay (RIPA) lysis buffer and separated by 10% sodium dodecyl sulfate–polyacrylamide gel electrophoresis (SDS PAGE), and then transferred onto polyvinylidene difluoride membranes. After blocking in 5% bovine serum albumin for 1 h at 37°C, the membranes were incubated with various antibodies (Abcam, MA, USA) at 4°C overnight. Sequentially, the membranes were incubated with the secondary antibody, and the proteins were visualized via electrochemiluminescence. Cells were seeded into a 60-mm plate at 4 × 10^5^ cells per plate for 48 hours. Cell lysates were subjected to sodium dodecyl sulfate–polyacrylamide gel electrophoresis, transferred to a nitrocellulose membrane, and immunoblotted with antibodies against Rabbit anti-human DMBT1 antibody and rabbit anti-humanC-IAP1 antibody (Cell Signaling Technology, CA, USA); antibodies of PI3K-AKT pathway which include: MMP-9, JUK, P-JUK, ERK, P-ERK, AKT, P-AKT(Cell Signaling Technology, CA, USA); normal mouse or rabbit IgG (Santa Cruz Biotechnology, Santa Cruz, CA, USA); mouse anti-FLAG, mouse anti-G FP (Sigma, CA, USA). The secondary anti-rabbit antibody was from GE Healthcare (Freiburg, Germany).β-actin (Cell Signaling Technology, CA, USA) served as a loading control.

### RNA isolation, quantitative real-time RCR (qRT-PCR)

Total RNA from tissues and cells was extracted by using TRIzol reagent (Invitrogen) according to the manufacturer's protocol. The RNA concentration was measured with a NanoDrop 2000 spectro-photometer (Thermo Fisher Scientific, USA). Total RNA (500 ng) was reverse-transcribed and amplified using the Applied Biosystems (Foster City, CA). qRT-PCR assays were performed by using PrimeScript RT Master Mix (Takara, Dalian, China) on the Applied Biosystems 7500 Real-time PCR System (ABI, USA). RNA from normal human tissues was purchased from Clontech (Palo Alto, CA). All reactions were run in triplicate and the comparative cycle threshold (CT) method was applied to quantify the expression level of CRNDE. Results were normalized to the expression of β-actin. For DMBT1 qRT-PCR, the primer pair 5′-ATTGTGCTGCACCTGGTCAT-3′ (forward) and 5′-AGCGGGAAGAGGGGTCATA-3′ (reverse) was used to amplify a 263-bp product. Human β-actin was using primers 5′-TTCACCACCATGGAGAAGGC-3′ (forward) and 5′-TGCATGGACTGTGGTCATGA-3′ (reverse) as the loading control. The relative amount of CRNDE was calculated using the equation2^−ΔΔCT^.

### siRNA construction and plasmid transfection

The small interfering RNA (siRNA) sequences targeting DMBT1, CRNDE and c-IAP1 and negative control were synthesized by Invitrogen (Shanghai, China) with the siRNA sequences listed in Supplementary Table S1. The DMBT1, CRNDE and c-IAP1 full-length sequence was synthesized and sub-cloned into a pWPXL vector (Invitrogen, Shanghai, China). Cells were cultured and transfected with si-RNAs or si-NC by using Lipofectamine 2000 (Invitrogen) and transfected with pWPXL vectors by using Lipofectamine 3000 (Invitrogen, Shanghai, China) according to the manufacturer's instructions. After transfection at different time point, cells were harvested for following analyses. The efficiency of knockdown and overexpression were determined by real-time quantitative reverse transcription-polymerase chain reaction (qRT-PCR).

### Cell proliferation assay

The Cell Counting Kit 8 (CCK-8, Dojindo, Kumamoto, Japan) was used to assess the relative cell viability at 24, 48, and 72 h after transfection according to the manufacturer's instruction. As for the colony formation assay, a total of 500 cells were seeded in 6-well plates and maintained in a medium containing 10% FBS for 2 weeks. The number of colonies was counted after fixing and staining for 20 min.

### Cell invasion assay

The cell invasion assay was performed with Transwell chambers (8-μm pore size; Millipore, MA, USA). A total of 2 × 10^5^ cells were seeded in the upper chamber coated with Matrigel for invasion assays. The upper chamber was filled with 200 μL of serum-free medium, while 800 μL of the medium with 10% FBS was added in the lower chamber. After incubation for 24 h, the cells on the filter surface were fixed and stained, followed by visualization using a phase-contrast inverted microscope.

### *In vivo* tumor formation assay

4 weeks male athymic BALB/c nude mice were maintained under specific pathogen-free conditions and manipulated according to protocols approved by the Shanghai Medical Experimental Animal Care Commission. Sh-DMBT1 or empty vector stably transfected GBC-SD cells were harvested. For tumor formation assay, 1 ×10^7^ cells was subcutaneously injected into a single side of each mouse. Tumor growth was examined every three days, and tumor volumes were calculated using the equation V = 0.5 × D × d^2^ (V, volume; D, longitudinal diameter; d, latitudinal diameter).

### Immunohistochemistry

All sections were dewaxed in xylene and rehydrated in graded ethanol, followed by incubating in 3% hydrogen peroxide for 10 min to quench endogenous peroxides. Samples were heated in 0.01 mol/L citrate buffer for 15 min at 100°C, and then put at room temperature for 30 min. After cooled, samples were blocked with 2% normal goat serum in PBS for 30 min to block antigenic epitopes, and then incubated with primary antibody (1:500 dilution) at 4°C overnight. After that, the sections were washed with PBS for three times, and then incubated with system-labeled HRP anti-mouse secondary antibody at room temperature for 20 min. Next, the sections were incubated in DAB and counterstained in Mayer's hematoxylin, dehydrated in alcohol and xylene. PBS was used as negative control. The score of the immunohistochemistry staining was evaluated by one investigator who was blinded to this study.

### Immunoprecipitation

GBC-SD cells in 60-mm dishes were washed with PBS and removed using a cell scraper and centrifuged at 1,2000 g for 30 min at 4°C. Lysate RIPA buffer was purchased from Beyotime (Shanghai, China). The sample was added with 1 μg antibodies by overnight incubations at. Protein-A agarose beads (30 μl) (Santa Cruz Biotechnology, USA) were added to the lysate, and the mixture was incubated under shaking at 4°C for 1 h. The beads were collected using centrifugation and washed three times with RIPA buffer. Proteins binding to the beads were eluted by adding 2 × sample treatment buffer boiling in 100°C for 10 mins for western-blot.

### Statistical analysis

The experimental data were analyzed by using Graphpad Prism 5 software (Graphpad Software Company, USA). The differences between groups were estimated by Student's *t*-test, analysis of variance (ANOVA) or non-parametric Mann-Whitney *U* test, Fisher's exact test, as appropriate. The results are represented as means ± standard deviation (S.D.). Data are presented as mean ± SEM. Survival curve was constructed by the Kaplan-Meier method and examined with the log-rank test. Univariate and multivariate analyses were used according to Cox proportional hazard regression model. All the cellular experiments were performed three or four times. P-value less than 0.05 were considered as statistically significant.

## SUPPLEMENTARY MATERIALS TABLES




