# Supplementary material for: Long non-coding RNA CRNDE promotes gallbladder carcinoma carcinogenesis and as a scaffold of DMBT1 and C-IAP1 complexes to activating PI3K-AKT pathway
Source: Oncotarget. 2016 Sep 14;7(45):72833–44. doi: 10.18632/oncotarget.12023 (PMC5341947; doi:10.18632/oncotarget.12023)
Supplement: Supplementary file 1 [file oncotarget-07-72833-s001.pdf]

Long non-coding RNA CRNDE promotes gallbladder carcinoma carcinogenesis and as a scaffold of DMBT1 and C-IAP1 complexes to activating PI3K-AKT pathway

Supplementary Materials

Supplementary Table S1: Relationship between DMBT1 expression and clinicopathologic factors of patients with gallbladder cancer

Parameter	No. of patients	DMBT1(low)	DMBT1 (high)	*P* -value
Sex				0.3506
male	71	30	41	
female	25	12	13	
Age (yr)				0.1123
< 60	63	30	33	
≥ 60	33	20	13	
Tumor differentiation				0.0321
I	10	3	7	
II	60	10	50	
III	35	5	30	
Tumor size (cm)				0.0023
≤ 5	50	34	21	
> 5	46	24	22	
Differentiation grade				0.2120
Well-moderate	54	23	31	
Poor-undifferentiation	40	12	28	
T stage				0.0013
T1–T3	57	16	41	
T4	42	31	11	
Lymph node status				
Negative	32	10	22	0.0001
Positive	69	8	61	
Distant metastasis				0.2107
M0	48	30	18	
M1	52	22	30	
TNM stage				0.0014
I–II	57	27	30	
III–IV	43	21	22	
Lymphatic invasion				0.0070
Negative	20	3	17	
Positive	76	15	61	
Venous invasion				0.0811
Negative	50	28	22	
Positive	46	26	20	

Supplementary Table S2: Univariate analysis identifies factors influencing the overall survival rate of gallbladder cancer patients

Factors	HR	95% CT	*P* value
Sex	0.985	1.01–2.41	0.631
Age (> 60 vs.≤ 60)	1.521	0.824–1.8921	0.752
Tumor size (cm) > 5	4.113	2.15–12.36	0.0589
Tumor size (cm) < 5	2.117	0.98–2.86	0.0821
Differentiation grade	4.532	2.115–6.101	0.0012
T stage	1.245	0.6–3.11	0.0001
Lymph node status	2.01	1.03–4.52	0.056
Distant metastasis	5.13	2.102–9.02	0.0002
TNM stage (III vs. II vs. I)	0.921	0.364–2.117	0.23
Lymphatic invasion	3.2	1.10–6.58	0.0011
Venous invasion	0.354	0.117–6.04	0.651

HR: hazard ratio; CI: confidence interval; TNM: tumor–node–metastasis classifications.

Supplementary Table S3: Multivariate analysis identifies factors influencing the overall survival rate of gallbladder cancer patients

Factors	HR	95% CT	*P* value
Tumor size (cm) > 5 vs. ≤ 5	1.531	1.01–3.11	0.080
Differentiation grade	3.15	2.13–5.12	0.0012
T stage	1.02	0.36–3.20	0.0051
Lymph node status	2.10	0.96–4.09	0.089
TNMstage (III vs. II vs. I）	1.75	1.02–2.69	0.0021
Lymphatic invasion	0.89	0.79–2.13	0.0036
Venous invasion	1.01	0.885–2.613	0.389

HR: hazard ratio; CI: confidence interval; TNM: tumor–node–metastasis classifications.

Supplementary Excel S1: Mass spectrum results of CRNDE in GBC cancer cells. See Supplementary _Excel_ S1
